# Animal-related injuries in a resource-limited setting: experiences from a Tertiary health institution in northwestern Tanzania

**DOI:** 10.1186/1749-7922-8-7

**Published:** 2013-02-01

**Authors:** Japhet M Gilyoma, Joseph B Mabula, Phillipo L Chalya

**Affiliations:** 1 Department of Surgery, Catholic University of Health and Allied Sciences-Bugando, Mwanza, Tanzania

**Keywords:** Animal related injuries, Injury patterns, Outcome, Tanzania

## Abstract

**Background:**

Animal related injuries are a major but neglected emerging public health problem and contribute significantly to high morbidity and mortality worldwide. No prospective studies have been done on animal related injuries in our setting. This study was conducted to determine the management patterns and outcome of animal related injuries and their social impact on public health policy in the region.

**Methods:**

This was a descriptive prospective study of animal related injury patients that presented to Bugando Medical Centre between September 2007 and August 2011. Statistical data analysis was done using SPSS computer software version 17.0.

**Results:**

A total of 452 (8.3%) animal-related injury patients were studied. The modal age group was 21-30 years. The male to female ratio was 2.1:1. Dog-bites (61.1%) were the most common injuries. Musculoskeletal (71.7%) region was the most frequent body region injured. Soft tissue injuries (92.5%) and fractures (49.1%) were the most common type of injuries sustained. Only 140 (31.0%) patients were hospitalized and most of them (97.1%) were treated surgically. Wound debridement was the most common procedure performed in 91.2% of patients. Postoperative complication rate was 15.9%, the commonest being surgical site infections (SSI) in 55.1% of patients. SSI was significantly associated with late presentation and open fractures (P < 0.001). The overall median duration of hospitalization was 16 days. Patients who had severe injuries, long bone fractures and those with hemiplegia stayed longer in the hospital (P < 0.001). Mortality rate was 10.2% and was significantly high in patients with severe injuries, severe head injury, tetanus and admission SBP < 90 mmHg (P < 0.001). The follow up of patients was poor.

**Conclusion:**

Animal related injuries constitute a major public health problem in our setting and commonly affect the young adult male in their economically productive age-group. Measures towards prevention and proper treatment and follow up are important in order to reduce morbidity and mortality resulting from this form of trauma

## Background

Animal related injuries are a major but neglected emerging public health problem and contribute significantly to high morbidity and mortality worldwide [1-3]. Human injuries resulting from encounters with domestic and wild animals are increasingly common throughout the world, particularly as ecosystems change and humans encroach on previously wild land [4,5]. The growing human population in developing countries such as Tanzania has brought animals and humans into closer physical contact, and prompted higher rates of animal attacks on humans [5,6]. This appears increased during times of drought and decreased availability of crop food, as well as when humans venture off frequently used paths [5-7].

Animals can cause injuries by various mechanisms that include bite, sting, crush, gore, stomp, buck off, fall on, peck, or scratch. In addition to inflicting traumatic injuries, animals transmit numerous zoonotic infections [8,9]. The threat of animal attacks on people is still a huge medico-social problem as these attacks result in millions of injuries and thousands of deaths all over the world [9-11]. Fortunately, the majority of such injuries are minor. It is estimated that about 60% of animal attacks lead to such mild injuries that the ambulatory treatment is sufficient, or the injured do not call for medical help at all [12].

However, many injuries remain undocumented and many people die, primarily in third-world countries, before receiving adequate medical care [13]. Besides, the medical and financial costs from both fatal and non-fatal animal encounters have a significant impact on public health [8]. Animal bite wounds are generally considered dirty or contaminated, and their treatment is difficult because of the risk of infection, especially in extensive injuries [14-17].

The outcome of treatment of animal related injuries may be poor especially in developing countries due to late presentation, lack of advanced pre-hospital care system and trauma centers and ineffective ambulance system for transportation of patients from the site if injury to hospital continues to be an area of neglect that prevents optimal trauma care [18].

There is paucity of information in most developing countries on animal related injuries where greater emphasis has been placed on injuries related to Road traffic accidents, which are more common. Given the proximity of our northwestern Tanzania hospital to Lake Victoria, Lake Tanganyika, and the Serengeti National Park, and presentation of several patients attacked by both domestic and wild animals with variety of complex injuries, we thought it was necessary to report our experience in the management of animal related injuries, outlining the management patterns and outcome of animal related injuries and their social impact on public health policy in the region.

## Methods

### Study design and setting

This was a five year descriptive prospective study of animal related injury patients that presented to the Accident and Emergency of Bugando Medical Centre (BMC) between September 2007 and August 2011. Bugando Medical Centre (BMC) is a referral, consultant and teaching hospital for the Catholic University of Health and Allied Sciences-Bugando (CUHAS-Bugando) and other paramedics and it is located in Mwanza city in the northwestern part of the United Republic of Tanzania. It is situated along the shore of Lake Victoria and has 1000 beds. BMC is one of the four largest referral hospitals in the country and serves as a referral centre for tertiary specialist care for a catchment population of approximately 13 million people from neighboring. There is no trauma centre or established advanced pre-hospital care in Mwanza city as a result all trauma patients are referred to BMC for expertise management.

### Study subjects

The subjects of this study included all patients of all age group and gender that presented to BMC with animal related injuries during the study period. Patients who failed to give proper information and those who had no relative to consent for the study were excluded from the study.

Recruitment of patients to participate in the study was done at the A & E department. Patients were screened for inclusion criteria and those who met the inclusion criteria were, after informed consent to participate in the study, consecutively enrolled into the study.

Patients with severe injuries were first resuscitated in the A&E department according to Advanced Trauma Life Support (ATLS). From the A & E department, patients were taken into the surgical wards or the intensive care unit (ICU) from where necessary investigations were completed and further treatment was instituted. Patients with open wounds and those with evidence of abdominal visceral injuries were taken to theatre for surgical intervention. Severe head injury patients with evident of space occupying lesions were also taken to theatre for possible craniotomy or burr holes and evacuation of haematoma. The severity of injury was determined using the Kampala trauma score II (KTS II) [19]. Severe injury consisted of a KTS II ≤ 6, moderate injury 7-8, and mild injury 9-10. Patients with head injuries were classified according to Glasgow Coma Scale (GCS) into: severe (GCS 3-8), moderate (GCS 9-12) and mild (GCS 13-15). An initial systolic blood pressure (SBP) on each patient was also recorded on admission. Routine investigations including hematological (hemoglobin, blood grouping & cross-matching), biochemical (serum creatinine & serum electrolytes) and radiological (x-rays of the chest & abdomen, abdominal ultrasound and CT scan) were performed on admission.

Depending on the type of injury, the patients were treated either conservatively or by surgery. All patients were followed up till discharged or death. This information was collected using a pre-tested questionnaire. Included in the questionnaire were socio-demographic data (age, sex, education and occupation), mechanism of injury, prehospital care, injury-arrival interval, admission haemodynamic parameters (e.g. systolic blood pressure and pulse rate), type and pattern of injury, trauma scores, body region injured, treatment offered, complications of treatment. Outcome variables were length of hospital stay, mortality and disability.

### Statistical data analysis

Statistical data analysis was done using SPSS software (Statistical Package for the Social Sciences, version 17.0, SPSS Inc, Chicago, Ill, USA). Data was summarized in form of proportions and frequent tables for categorical variables. Continuous variables were summarized using means, median, mode and standard deviation. P-values were computed for categorical variables using Chi-square (χ2) test and Fisher’s exact test depending on the size of the data set. Independent student *t*-test was used for continuous variables. Multivariate logistic regression analysis was used to determine predictor variables that are associated with outcome. A p-value of less than 0.05 was considered to constitute a statistically significant difference.

### Ethical considerations

The study was carried out after the approval by the department of surgery and BMC/CUHAS-Bugando ethics review board. An informed written consent was sought from patients or relatives.

## Results

### Socio-demographic data

During the period of study, a total of 54940 trauma patients were seen at BMC. Of these, 452 patients representing 8.3% of all trauma admissions had animal related injuries and these made the study population. The age of patients at presentation ranged from 9 to 86 years with a median age of 28 years. The peak age incidence was in the 21-30 years age group accounting for 248 (54.9%) patients. Males were 304 (67.3%) and females were 148 (32.7%), giving a male to female ratio of 2.1:1. Most of patients, 376 (83.2%) had either primary or no formal education and more than eighty percent of them were unemployed. Peasants and fisherman were the majority of animal related injury victims accounting for 302 (66.8%) and 100 (22.1%) patients respectively. The remaining 50 (11.1%) patients were school children, housewife or civil servants. The majority of patients, 322 (71.2%) came from the rural areas located a considerable distance from Mwanza City and more than ninety percent of them had no identifiable health insurance.

### Circumstances of the injury

The vast majority of patients, 356 (78.8%) sustained blunt injuries and the remaining 96 (21.2%) patients had penetrating injuries. The blunt to penetrating injuries ratio was 3.7: 1. The most prominent injuries were due to domestic animals accounting for 71.2% of cases (Table 1). Of the domestic animal related injuries, dog-bites were the most common injuries and were found to be greater in children compared to adults (p < 0.001), whereas other injuries were greater in adults than in children (p < 0.001).

**Table 1:** Distribution of animal related injuries according to animal species

**Animal species**	**Mechanism of injury**	**Number of patients**	**Percentage**
**Domestic animals**		**322**	**71.2**
· Dog	Bite, scratches	276	61.1
· Cow	Attacking with horns	15	3.3
· Cats	Bite, scratches	9	2.0
· Donkey	Kicks, fall	7	1.5
**Snake**	**Bite, Invenomation**	**62**	**13.7**
**Wild animals**		**31**	**6.9**
· Hyena	Bite, scratches	12	2.7
· Leopard	Bite, scratches	9	2.0
· Elephant	knocking over, Attacking with horns, battering	5	1.1
· Vervet monkey	Bite	4	0.9
· Lion	Bite	1	0.1
**Aquatic animals**		**7**	**1.5**
· Crocodiles	Bite, crush	6	1.3
Hippopotamus	Bite, knocking over	1	0.2
**Insects**	**Sting**	**16**	**3.5**
**Unspecified animal**	**Bite, scratches etc**	**14**	**0.9**

Following the injury events, none of the patients received any pre-hospital care and majority of them (382, 84.5%) were brought to the A & E department by relatives, friends or Good Samaritan, 67 (14.8%) by police and only three (0.8%) patients were brought in by ambulance.

### Injury characteristics

Musculoskeletal (extremities) region was the most common body region injured affecting 71.7% of patients (Table 2). Isolated injuries occurred in 402 (88.9%) patients while 50 (11.1%) patients had multiple injuries. Open wounds (i.e. bruises, abrasions, lacerations, punctured, avulsion, crush wounds etc) and fractures were the most common type of injuries sustained accounting for 92.5% and 49.1% of cases respectively (Table 3). Allergic reactions caused by insect stings were recorded in four patients.

**Table 2:** Site of injuries among the victims

**Site of injury**	**Number of patients**	**Percentage**
Musculoskeletal (extremities)	324	71.7
· *Lower limbs*	*(192)*	*(59.3)*
· *Upper limbs*	*(132)*	*(40.7)*
Abdomen	118	26.1
Chest	89	19.7
Head	76	16.8
Pelvis	17	3.8
Spines	12	2.7
Genitalia	9	1.9

Note: Some patients had more than one site of injuries.

**Table 3:** Distribution of patients according to type of injuries

**Type of injury**	**Frequency**	**Percentage**
Open wounds	418	92.5
Fractures	222	49.1
Visceral abdominal injuries	46	10.2
Intracranial hemorrhages	34	7.5
Pneumohemothorax	12	2.7
Traumatic amputations	10	2.2
Other minor injuries	23	5.1

According to Kampala Trauma Score II (KTS II) (Table 4), the majority of patients sustained mild injuries (KTS II = 9-10) in 312 (69.0%). moderate injuries (KTS II = 7-8) and severe injuries (KTS II ≤ 6) were recorded in 82 (18.2%) and 58 (12.8%) patients respectively.

**Table 4:** Kampala Trauma score

	**Description**	**Score**
**A**	**Age (in years)**	
	5-55	1
	< 5 or > 55	0
**B**	**Systolic blood pressure on admission (mmHg)**	
	< 89	2
	89-50	1
	>49	0
**C**	**Respiratory rate**	
	9-29/minutes	2
	>30/minutes	1
	≤ 9/minutes	0
**D**	**Neurological status**	
	Alert	3
	Responds to verbal stimuli	2
	Responds to painful stimuli	1
	Unresponsive	0
**E**	**Score for serious injury**	
	None	2
	One injury	1
	More than one injury	0

Kampala Trauma Score II total = A+B+C+D+E.

Interpretation.

KTS II < 6 = Severe injury.

KTS II 7-8 = Moderate injury.

KTS II 9-10 = Mild injury.

The Glasgow coma scale (GCS) indicated that 76 (16.8%) patients sustained head injuries. Of these, 41 (54.0%) sustained mild head injury, 20 (26.3%) patients sustained moderate head injury and 15 (19.7%) patients had severe head injury. The majority of patients, 398 (88.1%) had systolic blood pressure (SBP) > 90 mmHg on admission and the remaining 54 (11.9%) patients had SBP of 90 mmHg and below.

### Admission patterns and treatment

Most of patients (296, 65.5%) reported within 24 hours after injury. The time interval between injury and arrival to the A & E department ranged from 2 hours to 5 days with a median of 22 hours. The waiting time, defined as the time interval taken from reception at the A & E department and reception of treatment ranged from 30 minutes to 10 hours with a median of 3.00 hours. The majority of patients, 302 (66.8%) were attended to within 6 hours of arrival to the A & E department. Most of animal related injuries, 312 (69.0%) were so mild that after conservative (non-surgical) treatment (such as wound dressing, antibiotics, analgesics, tetanus toxoid, antirabies etc) at the A & E department the patients were discharged home. Only 140 (31.0%) patients were hospitalized. Of these, 102 (72.9%) were admitted to the surgical wards and the remaining 38 (27.1%) were admitted to the intensive care unit (ICU). All patients were administered antibiotics of varying nature at the A and E department. Analgesics (parenterally or orally) were also given to all patients. Four hundred and forty (97.3%) patients received tetanus toxoid and ninety-six (21.2%) patients received antirabies. Blood transfusion was given to twenty-one (4.6%) patients. The majority of patients (136, 97.1%) who were hospitalized were treated surgically. Wound debridement was the most common procedure performed in 91.2% of patients (Table 5).

**Table 5:** Type of surgical procedures performed (N= 136)

**Type of surgical procedures**	**Frequency**	**Percentage**
Wound debridement	124	91.2
Treatment of fractures	89	65.4
Exploratory laparotomy	46	33.8
Craniotomy ± burr holes/Elevation of depressed skull fractures	30	22.1
Limb amputation	28	20.6
Skin grafting/flaps	25	18.4
Pleural cavity drainage	12	8.8
Other surgical procedures	8	5.9

### Outcome and follow up of patients

A total of 98 complications were recorded in 72 (15.9%) patients the commonest being surgical site infections in accounting for 55.1% of patients (Table 6). The majority of patients (34, 63.0%) had polymicrobial bacterial profile. *Staphylococcus aureus* was the most common organism isolated accounting for 59.3% of all the bacterial isolates. According to multivariate regression logistic analysis, surgical site infections was significantly high in patients who presented late to the hospital (>24 hours) and those with open fractures (P < 0.001).

**Table 6:** Distribution of patients according to treatment complications (N= 98)

**Treatment complications**	**Frequency**	**Percentage**
Surgical site infections	54	55.1
Complications of fractures	38	38.9
Peritonitis/abdominal abscess	6	6.1
Complications of amputation stump	5	5.1
Tetanus	5	5.1
Skin grafting failure	2	2.1
Empyema thoracis	1	1.0
Post-traumatic epilepsy	1	1.0
Brain abscess	1	1.0

The overall length of hospital stay (LOS) for in-patients ranged from 1 day to 138 days with a median of 16 days. The LOS for non-survivors ranged from 1 day to 16 days (median 5 days). The length of ICU stay ranged from 1 to 18 days (median 4 days). Patients who had severe injuries (KTSII < 6), long bone fractures and those with hemiplegia secondary to spinal injuries stayed longer in the hospital (P < 0.001).

Out of 452 patients, 406 (89.8%) were alive and the remaining forty-six patients died in hospital giving a mortality rate of 10.2%. According to multivariate regression logistic analysis, mortality rate was significantly high in patients with severe injuries (KTSII < 6), severe head injury, tetanus and admission SBP < 90 mmHg (P < 0.001).

Of the survivors, 370 (91.2%) patients were discharged well, 5 (1.2%) patients were discharged against medical advice (DAMA) and the remaining 31 (7.6%) patients were discharged with permanent disabilities related to limb amputations, fracture complications, spinal cord injuries with neurological deficit. Only ninety-eight (21.7%) patients were available for follow-up at 6–12 months and the remaining patients were lost to follow-up.

## Discussion

In this review, animal related injuries occurred in 8.3% of all trauma admissions, a figure which is significantly higher than that reported by Moini *et al*[20] in Iran and Nogalski *et al*[11] in Poland. These differences in the rate of animal related injuries reflect differences in risk factors for animal related injuries between the study settings. The high figure of animal related injuries in this study may be due to the large number of patients with mild injuries which needed only ambulatory treatment and discharged.

The rate of the animal related injuries in the present study may be underestimated due to unreported patients, patients who died at scene or who did not reach our hospital because of treatment of minor injuries in private hospitals. A better picture of the magnitude of animal related injuries in our setting requires comprehensive data including police records, hospital admissions, and mortuary records. Better data could support useful policy guidance and help abate these injuries and their related morbidity and mortality.

In agreement with other studies [11,18,20], animal related injuries in our series were found to be most common in the third decade of life. High occurrences of animal related injuries among this age group have been attributed to a wide range of activities engaged in by this class of people. They represent the active group that partakes in high risk-taking activities such as farming, fishing, hunting, butchers, zoo and circus workers. The fact that this group represents economically productive age-group demands an urgent public policy response.

In our study, males were more affected than females, a finding which is in agreement with other studies [11,18,20,21]. Male predominance in the present study probably reflects the greater exposure of males to outdoor activities such as farming, fishing and hunting. Identification of risk taking behavior among trauma patients has potential significance for the prevention of injuries.

The majority of patients in this study came from the rural areas located a considerable distance from the study area. This is in contrast to Moini *et al*[20] who reported that animal related injuries affected both rural and urban dwellers. Farmers in rural areas are at high risk of being attacked by either wild, domestic, aquatic animals or snakes.

Previous studies conducted in the United States of America reveal that animals are one of the main causes of injuries in the farming industry [22,23], which is similar to what was found in our series. This observation is at variant with Moini *et al*[20] who reported that animal-related injuries were more common in house wives than farmers. The finding that more than eighty percent of victims of this form of trauma had no definable source of private or governmental health care insurance at the time of their injury calls for urgent public policy response.

The prehospital care of trauma patient has been reported to be the most important factor in determining the ultimate outcome after the injury [24]. None of our patients had pre-hospital care; as a result the majority of them were brought in by relatives, Good Samaritan and police who are not trained on how to take care of these patients during transportation. The lack of advanced pre-hospital care and ineffective ambulance system for transportation of patients to hospitals are a major challenges in providing care for trauma patients in our environment and have contributed significantly to poor outcome of these patients.

Late presentation following injury is a common phenomenon in most developing countries including ours and is usually associated with increased rate of complications [18]. The majority of our patients presented early within 24 hours of their injuries. This finding is in agreement with other studies [18,25]. Early presentation in our study reflects the low complication rate in our patients.

In our study, dog bite was the most common cause of injuries and commonly affected children more than adult. This finding is in agreement with several studies that indicated dogs as the primary animal species implicated in animal related injuries ranging from 63-80% [26], but contrary to other studies which reported that equestrian traumas are common [27,28]. Higher dog attacks in children are thought to be attributable to their size and the proximity of their face to the dogs’ mouth, and these attacks are generally related to the children’s interaction with the dog, possibly provoking the attack [29].

Given the proximity of our northwestern Tanzania hospital to Lake Victoria, Lake Tanganyika, and the Serengeti National Park, one would expect large number of injuries resulting from aquatic and wild animals. The low rate of injuries from aquatic and wild animals in our study can be explained by the fact that the majority of patients sustaining these severe injuries may have died before reaching the Accident and Emergency department. These animals can produce severe injuries by grasping victims with their powerful jaws and dragging them underwater, where they roll while crushing their prey [18].

In keeping with other studies [30,31], the majority of injuries in the present study were in the lower and upper limbs. Attempts at using, the foot and hand to avoid animal bite may be the possible reason for these parts being affected more. The other thought is that animals may be at ease to attack moving body parts [14,15,31].

The type of wounds in injuries resulting from animal attack can range from minor bruises to more extensive injuries like punctured wounds, avulsions, amputations and separation of a pedunculated flap [18,32]. In this study, open wounds such as bruises, abrasions, lacerations, punctured, avulsion, crush wounds etc and fractures were the most common type of injuries sustained. Limb amputation was reported in only 2.2% of cases mainly due to large wild and aquatic animals. Similar observation was reported previously by Chalya *et al*[18] at the same institution.

It has been estimated that about 60% of animal attacks lead to such mild injuries that the ambulatory treatment is sufficient, or the injured do not call for medical help at all [22,33]. The majority of patients in our series sustained mild injuries which is comparable with other studies [18,22]. The large number of patients with mild injuries in this study may be responsible for the low rate of hospitalization and complications among these patients.

The principles of management of wounds resulting from animal attacks include cleaning and debriding the wound, consideration of prophylactic antibiotics, treatment of infectious complications when they develop and appropriate use of tetanus vaccination [17,18,32]. Whereas minor wounds are usually treated conservatively with prophylactic antibiotics, analgesics, tetanus toxoid and cleaning of wounds with normal saline and apply dressing, extensive wounds require operative procedures mainly debridement and primary or delayed primary closure.

In our study, the vast majority of hospitalized patients were treated surgically and surgical wound debridement with either primary or delayed closure was the most frequent surgical procedure performed.

In this study, wound infection was the most common complication and majority of patients had polymicrobial bacterial profile. *Staphylococcus aureus* was the most common organism isolated. Similar observation was also noted previously at the same study area by Chalya *et al*[18] reflecting no change of bacterial profile in this region.

The current study had a mortality rate of 10.2%, which is higher than that reported by others [20]. High mortality rate in our study was recorded in patients with severe injuries, severe head injury, tetanus and shock on admission.

The length of hospital stay (LOS) has been reported to be an important measure of morbidity among trauma patients. Prolonged hospitalization is associated with an unacceptable burden on resources for health and undermines the productive capacity of the population through time lost during hospitalization and disability. Our figures for the overall median LOS in the present study were higher than that reported by others [11,20,31]. Patients who had severe injuries, long bone fractures and those with hemiplegia secondary to spinal injuries stayed longer in the hospital. However, due to the poor socio-economic conditions in Tanzania, the duration of inpatient stay for our patients may be longer than expected.

Generally, the overall outcome of our patients was good as more than ninety percent of patients (survivors) were discharged well without permanent disabilities.

Self discharge by patient against medical advice is a recognized problem in our setting and this is rampant, especially amongst trauma patients [34]. Similarly, poor follow up visits after discharge from hospitals remain a cause for concern. These issues are often the results of poverty, long distance from the hospitals and ignorance. Delayed presentation, inadequate ICU space, discharge against medical advice, and the large number of loss to follow up were the major limitations of this study. Another potential limitation was that the analyzed group of patients was treated at a single medical centre. For that reason, the results may not be adequate for the whole population in this part of Tanzania. However, despite these limitations, the study has provided local data that can be utilized by health care providers to plan for preventive strategies as well as establishment of management guidelines for patients with animal related injuries. The study also provides a comparable data to the other parts of the world in the field of animal related injuries. The challenges identified in the management of these patients in our setting need to be addressed, in order to deliver optimal trauma care for the victims of animal related injuries.

## Conclusion

Animal related injuries in this region affect predominantly young adult males in their economically productive age - group. The severe injury group requires great hospital resources and show high morbidity, mortality and permanent disability. Thus constituting a major health regional problem, they require closer observation and analysis from the decision makers to provide appropriate health promotion and prevention measures as well as assuring great resources for their proper treatment.

## Competing interests

The authors declare that they have no competing interests.

## Authors’ contributions

JMG conceived the study, participated in the design and coordination of the study and drafted the manuscript. JBM and PLC contributed in study design, literature search, data analysis, manuscript writing and editing. In addition PLC submitted the manuscript. All the authors read and approved the final manuscript.
